# Optical Detection of Paraoxon Using Single-Walled Carbon Nanotube Films with Attached Organophosphorus Hydrolase-Expressed *Escherichia coli*

**DOI:** 10.3390/s150612513

**Published:** 2015-05-27

**Authors:** Intae Kim, Geon Hwee Kim, Chang Sup Kim, Hyung Joon Cha, Geunbae Lim

**Affiliations:** 1 Department of Mechanical Engineering, Pohang University of Science and Technology, 77 Cheongam-Ro, Nam-Gu, Pohang, Gyeongbuk 790-784, Korea; E-Mails: one@postech.ac.kr (I.K.); q16164545@postech.ac.kr (G.H.K.); 2 School of Biotechnology and Graduate School of Biochemistry, Yeungnam Univerisity, 280 Daehak-Ro, Gyeongsan, Gyeongbuk 712-749, Korea; E-Mail: cskim1409@ynu.ac.kr; 3 Department of Chemical Engineering, Pohang University of Science and Technology, 77 Cheongam-Ro, Nam-Gu, Pohang, Gyeongbuk 790-784, Korea; E-Mail: hjcha@postech.ac.kr

**Keywords:** organophosphates, biosensor, carbon nanotube, microbial immobilization

## Abstract

In whole-cell based biosensors, spectrophotometry is one of the most commonly used methods for detecting organophosphates due to its simplicity and reliability. The sensor performance is directly affected by the cell immobilization method because it determines the amount of cells, the mass transfer rate, and the stability. In this study, we demonstrated that our previously-reported microbe immobilization method, a microbe-attached single-walled carbon nanotube film, can be applied to whole-cell-based organophosphate sensors. This method has many advantages over other whole-cell organophosphate sensors, including high specific activity, quick cell immobilization, and excellent stability. A device with circular electrodes was fabricated for an enlarged cell-immobilization area. *Escherichia coli* expressing organophosphorus hydrolase in the periplasmic space and single-walled carbon nanotubes were attached to the device by our method. Paraoxon was hydrolyzed using this device, and detected by measuring the concentration of the enzymatic reaction product, *p*-nitrophenol. The specific activity of our device was calculated, and was shown to be over 2.5 times that reported previously for other whole-cell organophosphate sensors. Thus, this method for generation of whole-cell-based OP biosensors might be optimal, as it overcomes many of the caveats that prevent the widespread use of other such devices.

## 1. Introduction

Organophosphates (OPs) are widely employed in the agricultural industry as insecticides, however, their long-term toxicity toward both humans and the ecosystem is an important issue worldwide. OP pesticides deactivate the enzyme AChE, which is essential for nerve function in insects and animals, including humans [1,2]. Additionally, OPs are absorbable by all routes, including inhalation, ingestion, and through the dermis. Therefore, the detection and sensing of OPs is important for safety. Traditional methods, such as gas chromatography, high-performance liquid chromatography, and mass spectroscopy, and biological methods, such as immunoassays, have been used for the analysis of OPs [3,4]. However, such analyses are generally performed in the lab rather than in the field, require expensive analytical instruments and expertise, involve several sample preparation steps, and are time-consuming [5]. To overcome these shortcomings, a sensor that is fast and inexpensive with the ability of on-site detection is needed.

Organophosphorus hydrolase (OPH) hydrolyzes a wide range of OP compounds that are used as pesticides and chemical warfare agents. OPH can hydrolyze P-O, P-S, P-F, and P-CN bonds, resulting in a change in pH [6,7,8,9,10,11,12]. Hydrolysis products can be analyzed by spectrophotometry or electrochemical detection methods. Because the rate of product formation is proportional to the concentration of OP compound, the OP concentration can be determined by measuring the hydrolysis product [13]. This method has the following advantages over inhibition-based methods: (1) it does not require a continuous supply of the enzyme substrate, because the analyte itself is the substrate; and (2) enzymes such as AChE are inhibited by both OP and carbamate pesticides, rendering OPH more selective, as it only detects OPs [5,14].

Microbes are a common host for enzyme production. However, to obtain usable enzymes, the expressed enzymes need to be purified and, oftentimes, enzymes are expressed in the intracellular space as inclusion bodies. Recently, the secretion of enzymes into the periplasmic space or cell surface has been demonstrated [15,16]. With this technique, enzymes in the periplasmic space or cell surface can come into contact with reactants without the need for a purification process and, thus, microbes can be used as whole-cell catalysts to replace the use of purified enzymes [17,18,19,20,21]. Microbes that express OPH in the periplasmic space or cell surface have also been developed [15,19,22,23,24], and electrochemical sensors using *Escherichia coli (E. coli)* with surface-expressed OPH have been reported [17,18]. An optical microbial biosensor for the detection of OP compounds using *E. coli* expressing OPH in the periplasmic space has also been reported [21].

A whole-cell biosensor meets the requirements of a convenient OP-sensing tool, and is both more stable and less expensive than enzyme-based biosensors. Amperometric, potentiometric, and spectrophotometric detection methods have been used with OPH-expressing microbial biosensors. Spectrophotometric OP sensors measure the absorption wavelength of *p*-nitrophenol (PNP, 400 nm), which is produced by the hydrolysis of OP compounds [25,26,27,28]. Optical methods are advantageous in that they are not affected by electric and magnetic fields, have excellent selectivity, and can be manufactured to be portable by miniaturization of spectrophotometers. The detection limit of these methods using a standard sample container is approximately 5 µM, which, albeit higher than that of amperometric OP sensors, is sufficient for field usage (the rat oral LD_50_ is 1800 μg/kg, or 6.54 μM·L/kg) [29].

To use whole-cells as a biocatalyst, cells must be immobilized to ensure reusability by preventing their detachment. Various microbial immobilization methods, such as flocculation, adsorption, covalent bonding to a carrier, crosslinking of cells, encapsulation in a polymer gel, and entrapment in matrices or biofilms, have been devised and used in macro-scale systems [30,31,32]. We previously reported a microbe immobilization method, microbe-attached single-walled carbon nanotube (SWNT) film, for microelectromechanical system (MEMS)-based chips to apply microbes in a microfluidic system [33]. This process of microbe immobilization is simple and fast. The film is fabricated site-specifically and has a floating porous structure that enhances the efficiency of enzyme activity. This method is optimized to immobilize microbes over a small space at high density; however, it is not suited for large-scale (larger than several square centimeters) immobilization of microbes due to cost. Other whole-cell OP sensors use a standardized platform, either a 96-well microplate or a disposable cuvette, which have a 50- to 200-μL sample volume [21,25,26]. We expect that our method also has advantages over this sample volume range.

In the present paper, we demonstrate that microbe-attached SWNT film can be applied to whole-cell optical OP sensors and exhibits better performance compared to other whole-cell OP sensors. The device, which has a circular comb drive-shaped electrode, was fabricated for a large area of cell immobilization, and microbe-attached SWNT film was formed between electrodes using *E. coli* that express OPH in the periplasmic space. The concentration of paraoxon was measured using this device by spectrophotometry at 400 nm, and the advantages of this method were demonstrated by comparing it to other whole-cell OP sensors.

## 2. Experimental Section

### 2.1. Preparation of Carbon Nanotube (CNT) Solution

Acid treatment of CNTs by liquid-phase oxidation is a commonly used surface modification method for the introduction of carboxylic groups [34,35,36]. Both the metallic catalyst particles and the amorphous carbon particles are removed by acid treatment. Moreover, CNTs are dispersed by this process, because the introduced carboxylic groups repel each other and exhibit hydrophilic properties.

In this study, a SWNT solution was prepared by oxidation of SWNTs in a strong acid solution, with sonication to generate well-dispersed SWNTs [37,38]. SWNTs, produced by an arc discharge process, were purchased from Hanwha Chemical (Seoul, Korea). Five milligrams of SWNTs were suspended in concentrated H_2_SO_4_/HNO_3_ (50 mL, 3:1, v/v) and sonicated in an ultrasonic bath for 3 h. The SWNT suspension was diluted with deionized (DI) water (2 L) and collected on a membrane filter (Millipore, Darmstadt, Germany) by vacuum filtration. The collected SWNTs were resuspended with 50 mL DI water so that the pH of the suspension was neutral. After centrifuging at 9000 *g* for 5 min, sediments were removed.

**Figure 1:**
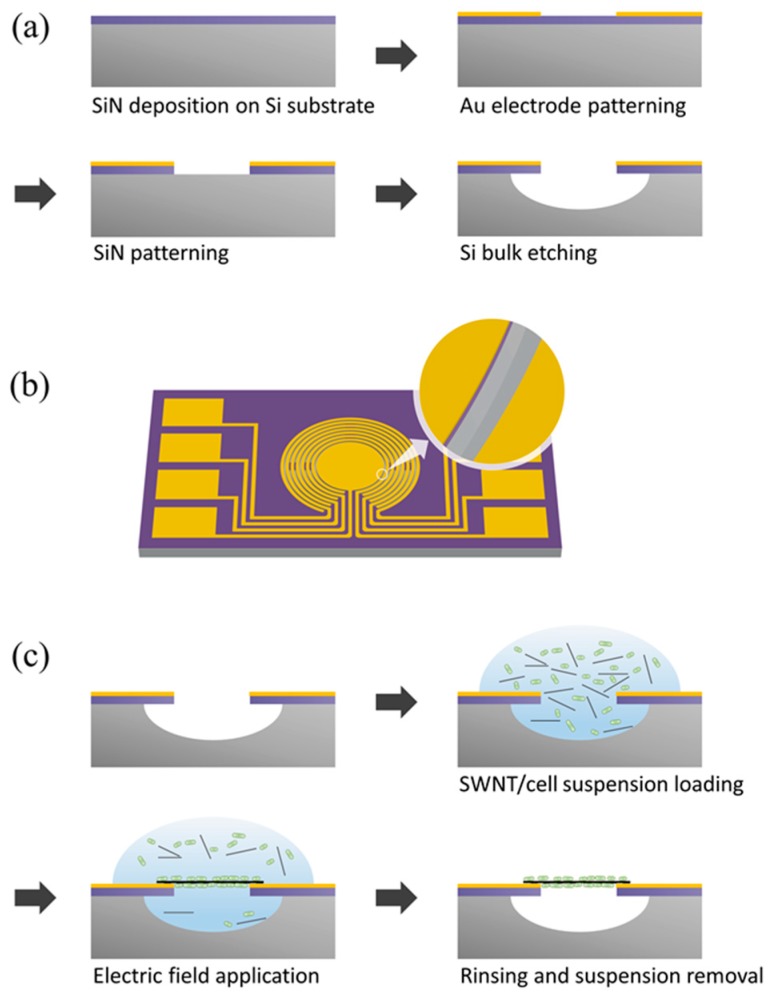
Device Preparation. Schematic representation for (**a**) the fabrication process of the device containing floating electrodes, (**b**) the complete device and an enlarged image of its bulk-etched gap, and (**c**) the fabrication process of *E. coli*-attached SWNT film.

### 2.2. Preparation of OPH-Expressing E. coli

Recombinant *E. coli* cells expressing both OPH in the periplasm and the chaperone GroEL/ES in the cytoplasm [21,23] were used as the target microorganism. Cells were cultured in Luria-Bertani (LB) medium (0.5% (w/v) yeast extract, 1% (w/v) tryptophan, and 1% (w/v) NaCl) containing 50 μg/mL ampicillin (Sigma-Aldrich, St. Louis, MO, USA) and 25 μg/mL chloramphenicol (Sigma-Aldrich). For efficient expression of recombinant OPH and GroEL/ES, 1 mM (final concentration) isopropyl-β-D-thiogalactopyranoside (IPTG; Sigma-Aldrich), 10 ng/mL tetracycline, and 0.5 mM CoCl_2_ were added when the culture reached a cell density (OD_600_) of 1.2. The recombinant cells were grown for an additional 12 h at 37 °C. The cells were harvested by centrifugation at 4000 *g* for 10 min. The harvested cells were resuspended in Tris-HCl buffer (pH 8.0) at various densities and diluted tenfold with SWNT suspension.

### 2.3. Fabrication Process for the Cantilever Electrodes

Figure 1a shows a schematic diagram of the fabrication process for the cantilever electrodes, which were fabricated using a MEMS. The cantilever structure was formed by depositing a low-stress nitride on a silicon (Si) substrate using low-pressure chemical vapor deposition (LPCVD). Gold (Au) electrodes were patterned on a silicon nitride (SiN) surface by a lift-off process (Cr/Au 20/200 nm). To construct the cantilever structure, the SiN was patterned by reactive-ion etching (RIE), and the Si was etched by isotropic wet etching. Lastly, an insulating layer was patterned on the Au electrodes and SiN surface using SU8 photoresist (MicroChem, Newton, MA, USA).

### 2.4. Fabrication of E. coli-Attached SWNT Film

Figure 1c shows the fabrication process of *E. coli*-attached SWNT film. Recombinant *E. coli* cells expressing OPH in the periplasm were used as a whole cell catalyst. The cell density of the suspension was adjusted to OD_600_ = 16. To prepare the *E. coli*-mixed SWNT suspension, the cell suspension and SWNT solution were mixed at a ratio of 1:9 (v/v). To fabricate the *E. coli-*attached SWNT film, an *E. coli*-mixed SWNT suspension was placed between the suspended electrodes, and an AC voltage with an amplitude of 7 V_PP_ and a frequency of 1 MHz was applied between the electrodes for 10 s. Then, SWNTs and *E. coli* were aligned between electrodes by dielectrophoresis force. The device was then rinsed in Tris-HCl buffer solution (pH 8.0, 10 mM).

### 2.5. Fabrication of Paraoxon-Sensing Platform

**Figure 2:**
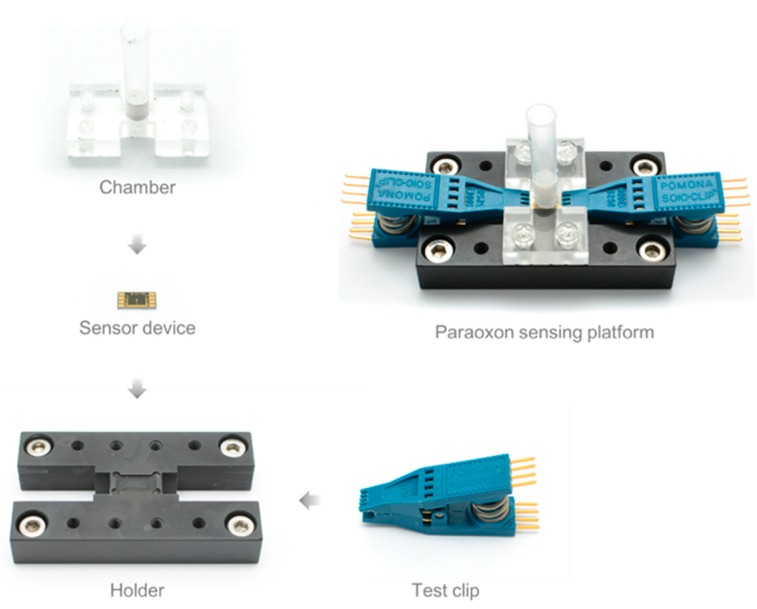
Fabrication process of a platform for a whole-cell OP sensor using *E. coli*-attached SWNT film.

The platform of a microbe-attached SWNT film biosensor consists of four parts (Figure 2): a sensor holder, a sensor device, a chamber, and a test clip. The sensor holder was made with polyether ether ketone (PEEK) by a mechanical machining process. PEEK exhibits excellent mechanical and chemical resistance properties that are retained at high temperature. Furthermore, PEEK is an excellent insulator. These properties are suitable for the sensor holder material. The sensor holder has a shallow trench to fix the sensor device, and is pitted on both sides to connect a test clip. A chamber is needed to place the sample to the sensor surface, in which *E. coli* is immobilized. The chamber consists of three parts: a body to connect the chamber to the sensor holder, a tube to store the sample, and a polydimethylsiloxane (PDMS) gasket to seal between the chamber and the sensor device. The chamber was designed for a 500 μL sample and is autoclavable. The test clip was adopted to allow for an easy connection between electrodes in the sensor device.

### 2.6. Paraoxon Measurement Setup

Paraoxon solution was prepared at various concentration (20–500 μM) in 100 mM 2-(*N*-cyclohexylamino)ethanesulfonic acid (CHES) buffer (pH 9.0; Sigma-Aldrich). Paraoxon is hydrolyzed to *p*-nitrophenol (PNP) and diethylphosphoric acid by OPH. Therefore, the catalytic reaction of OPH can be measured by determining PNP density. Paraoxon (500 μL) was added to the chamber (Figure 2) and reacted for 50 min at 25 °C. The PNP density of the solution was then determined using a spectrophotometer to measure the absorbance of the solution at 400 nm (Mecasys, Daejeon, Korea), which is the absorption wavelength of PNP produced by the hydrolysis of OP compounds.

## 3. Results and Discussion

### 3.1. E. coli-Attached SWNT Film Fabrication

For the spectrophotometric whole-cell based OP sensor, immobilization of a sufficient amount of cells is required. Our cell immobilization method requires two cantilever electrodes that have an appropriate gap size (5–10 μm) for inducing an even dielectrophoresis (DEP) force. The area of cells immobilized is proportional to the area between the two cantilever electrodes. In our previous study, we fabricated a device containing two electrodes of 20-μm width and a 5-μm gap between the two electrodes [33]. In this study, we designed eight cantilever electrodes that have a circular comb drive shape for a larger area of cell immobilization than in the previous device (Figure 2). Eight Au electrodes, having widths of 20 μm, were placed parallel to each other with a 5-μm gap between adjacent electrodes. Consequentially, the area of the gaps between electrodes that was able to form the microbe-attached SWNT film was 0.043 mm^2^, and the total area that contains the eight electrodes was 0.26 mm^2^.

**Figure 3:**
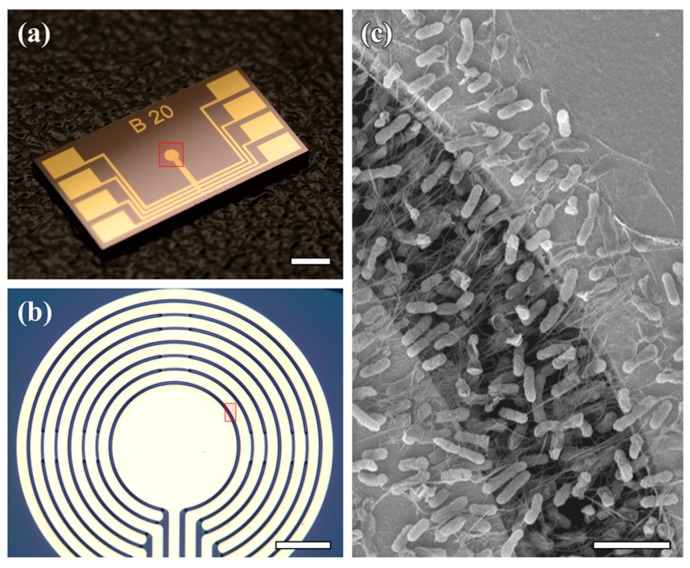
Device Imaging. Optical image of (**a**) the entire device (scale bar: 2 mm) and (**b**) a magnified view of the circular comb drive-shaped electrodes (scale bar: 50 µm). (**c**) SEM image of *E. coli-*attached SWNT film (scale bar: 5 µm). The red square in (a) indicates the image area of (b), and the red square in (b) indicates the image area of (c).

To fabricate an *E. coli-*attached SWNT film on the device, an AC voltage with an amplitude of 7 V_PP_ and a frequency of 1 MHz was applied between the two electrodes, across the mixed SWNT/*E. coli* suspension, for 10 s. This process was repeated in sequence from the innermost electrodes to the outermost electrodes to fabricate *E. coli-*attached SWNT film in every gap between the electrodes. Figure 3c shows a scanning electron microscopy (SEM) image of *E. coli*-attached SWNT film. Two cantilever electrodes are connected with SWNTs, and cells are attached to the SWNTs. Because the SWNTs are a one-dimensional structure, the attachment area of cells is minimized, while the reaction area of cells is maximized. In addition, *E. coli*-attached SWNT film is floated from the bottom of the substrate and contains spaces between cells that can pass substances. Therefore, mass transfer of the floated film is enhanced compared to the film attached to the floor of the substrate.

### 3.2. Structural Advantage of E. coli-Attached SWNT Film

For the optical density-based whole-cell OP sensor, it is advantageous to maximize the enzyme activity per unit area in which cells are immobilized. Our immobilization method has advantages in this aspect. First, cells can be immobilized at a high density. When the electric field conditions are 6 V_pp_ and 1 MHz over 5 s, and the *E. coli* density (OD_600_) is 0.2, the cell density of the film is 0.619 cells/ μm^2^ [33]. Although this value is not directly comparable with other studies, it is clear that our method can immobilize cells at a high density, considering the fact that the two-dimensional area of *E. coli* is about 1 µm^2^. Second, the efficiency of enzymatic activity is increased. This is because mass transfer is enhanced by the flotation from the substrate and porous structure of the film and the reaction area of the cell is maximized by attaching cells to SWNTs of a one-dimensional structure.

**Figure 4:**
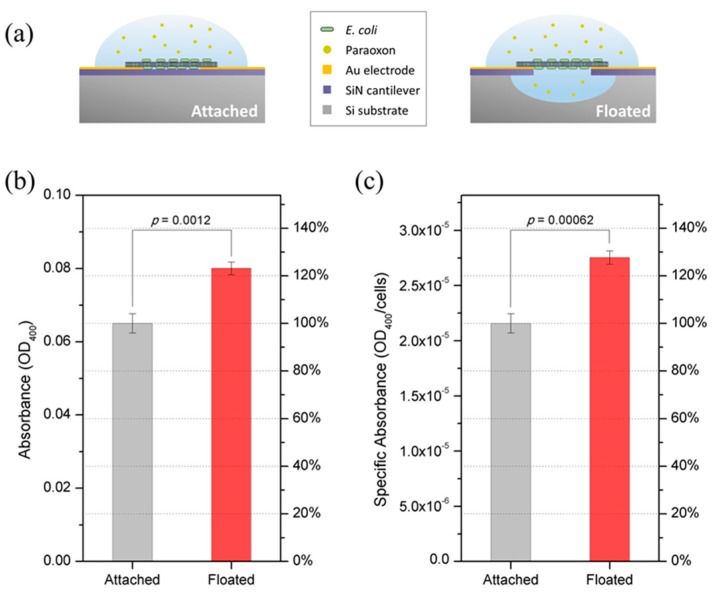
Device Activity. (**a**) Schematic representation of attached and floating *E. coli-*attached SWNT films. The floating film device has space for floating the *E. coli-*attached SWNT film from the surface of the device. On the other hand, the device for attached film did not undergo the etching process of silicon and silicon nitride to produce the film on the surface of the device; (**b**,**c**) show the comparison of enzymatic activity between the attached and floating films (b) per device and (c) per cell. Error bars indicate standard deviations.

To demonstrate the structural advantage of *E. coli-*attached SWNT film, two kinds of devices were prepared. They have the same electrode pattern, but the device for attached film did not undergo SiN etching and Si bulk etching in the fabrication process as did the device for floating film (Figure 4a). Each device was designed to have seven pairs of facing electrodes, with a width of 100 μm and a gap size of 5 um. To fabricate *E. coli-*attached SWNT film on the two devices, an AC voltage with amplitude of 7 V_PP_ and frequency of 1 MHz was applied between the two electrodes, across the mixed SWNT/*E. coli* suspension (OD_600_ = 0.4), for 5 s. Figure 4b,c show the activity of *E. coli*-attached SWNT films, comparing the one floated from the surface to the one attached on the surface of the substrate. *E. coli*-attached SWNT film that is floated showed activity that was improved by 23% over the attached one (Figure 4b). Because a greater number of cells are incorporated when the film is attached on the surface of the device compared to the floated one, the activity per cell was calculated. *E. coli*-attached SWNT film that is floated showed a 27% improvement in activity over the attached one (Figure 4c).

### 3.3. Paraoxon Measurement and Comparison with Other Whole-Cell OP Sensors

**Figure 5:**
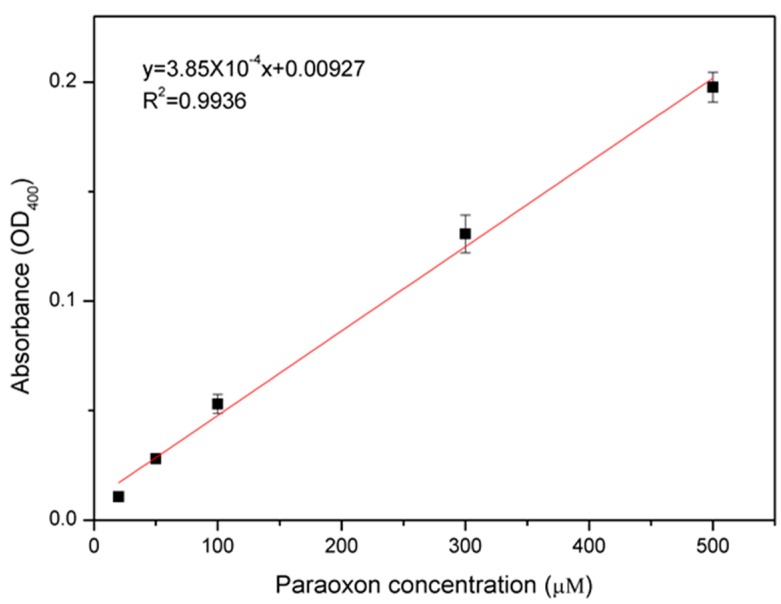
Detection of paraoxon using *E. coli*-attached SWNT film. Error bars indicate standard deviations. The absorbance increased linearly as the paraoxon concentration was increased.

We measured the paraoxon concentration using *E. coli*-attached SWNT film by a spectrophotometric method, and compared our results with other whole-cell based OP sensors. Paraoxon was detected by measuring absorbance at 400 nm. Each density of paraoxon solution (500 μL, 100 mM CHES buffer, pH 9.0) was reacted and measured. Figure 5 shows quantitative detection using various concentrations of paraoxon: 20, 50, 100, 300, and 500 μM. Error bars indicate standard deviations. The absorbance increased linearly as the paraoxon concentration was increased. The coefficient of determination has a high value (*R^2^* = 0.9936) in the tested range of paraoxon concentrations.

In the absorbance-based whole-cell biosensor, enzyme activity per unit area is an important factor related to sensor performance. High enzyme activity per unit area can lead to enhanced sensitivity, faster measurement time, and a reduction in sample volume. For comparison with other studies on whole-cell based OP sensors, we defined enzyme activity per unit cell-immobilized area as specific activity (μmol·min^−1^·mm^−2^) and calculated its value when the paraoxon concentration is 100 μM. Specific activity is defined as:

(1) $$Specific\;activity=c\times V\times \frac{1}{t}\times \frac{1}{a}$$

where c is the concentration of PNP, V is the sample volume, t is the enzymatic reaction time, and a is the area of immobilized cells. Because A=εcl (A is the absorbance, ε is the molar absorption coefficient, and l is the light path length) by the Beer-Lambert law, specific activity is conversed as follows:

(2) $$Specific\;activity=\frac{AV}{\varepsilon lta}$$

The ε of PNP is 17,000 M^−1^∙cm^−1^, and the other parameters are determined by the experimental methods of each study. Table 1 shows a comparison of whole-cell based OP sensors, including the one developed in this study, and our device shows the highest specific activity. The specific activity of our device shown in this study is more than 2.5 times the best previously reported result [25]. This is due to the ability of *E. coli*-attached SWNT film to immobilize cells at a high concentration and enhance the enzymatic activity due to structural advantages. Our immobilization method requires only 10 s for each pair of electrodes, and is able to be fabricated consecutively, so the required time for cell immobilization can be represented as: required time = (number of electrode pairs) ×10 s + (rinsing time). In this study, we used a device that has seven pairs of electrodes, so the total required time for cell immobilization is less than 100 s. The time for cell immobilization reported in other studies includes only the steps whose exact time duration is mentioned, and does not include the time required for drying, solution changing, and sample preparation and manipulation. In addition, our method exhibits the highest long-term storage stability. These results suggest that *E. coli*-attached SWNT film exhibits excellent performance, reliability, and a fast fabrication process compared to previously-reported cell immobilization methods for whole-cell based OP sensors.

**Table 1:** Characteristic comparison between this study and other whole-cell based OP sensors.

Immobilization Method	Cell Type	Substrate	Specific Activity (μmol·min^−1^·mm^−2^)	Time for Cell Immobilization	Long-Term Storage Stability	Reference
MAP-based adhesion	*E. coli* BL21	Paraoxon	19.06	>2 h	28 d (80%)	[21]
Glutaraldehyde-based chemical crosslinking	*Sphingomonas* sp. JK1	4(methyl parathion)	43.18	>1 h	18 d (80%)	[25]
Glutaraldehyde-based chemical crosslinking	*Sphingomonas* sp. JK1	4(methyl parathion)	43.12	>1 h	32 d (90%)	[26]
Adsorption by Van der Waals force	*E. coli* BL21	Paraoxon	119.91	<100 s	37 d (90%), 67 d (80%)	This study

Table 2 shows the immobilized cell area required to achieve the performance of previously-reported whole-cell based OP sensors [21,25,26] using typical spectrophotometric sensing platforms. The detection limit and time were chosen as 5 μM and 5 min, respectively, because other whole-cell OP sensors are 4–5 μM and 5 min, respectively. The absorbance was chosen as OD_400_ = 0.01, because the noise level of conventional spectrophotometers is 0.0001–0.001 at OD_400_ = 0.5. We calculated the required area of immobilized cells from Equation (2), and the specific activity was adjusted to the concentration of paraoxon, because specific activity is proportional to the concentration of substrate. Most absorbance-based analyses use a spectrophotometer or microplate reader. Commercially-available equipment and common sample containers were selected for Table 2. Disposable cuvettes and 96-well microplates are the most commonly used, because they allow for multi-sample analysis and are disposable, standardized, and cheap. The required area for a disposable cuvette is 1.374 mm^2^, and that for a 96-well microplate is 6.083 mm^2^. If a larger area is applied to cell immobilization, higher performance can be expected compared to other whole-cell OP sensors.

**Table 2:** The cell immobilization area required to detect 5 μM of paraoxon in 5 min using typical spectrophotometric sensing platforms.

Equipment	Conventional Spectrophotometer	Conventional Spectrophotometer	Microplate Reader	Microvolume Spectrophotometer
Sample container	cuvette (disposable)	cuvette (quartz)	96-well microplate	Directly on the equipment
Sample volume (μL)	70	50	200	0.5
Light path length (mm)	10	10	6.5	0.5
Required area (mm^2^)	1.374	0.981	6.038	0.196

## 4. Conclusions

In this study, we fabricated *E. coli*-attached SWNT film to immobilize cells on a MEMS-based chip, and demonstrated that *E. coli*-attached SWNT film can be applied as a spectrophotometric OP sensor. *E. coli*-attached SWNT film is a unique cell immobilization method for MEMS-based chips and has several advantages, including site-specificity, speed, and stability. As the sample volume is small, our method is the only way to immobilize microbes. However, the sample volume is greater than the approximately 50 μL that several methods, including chemical surface modification, crosslinking, and adhesive material coating, require. The important consideration is that the sample volume of 96-well microplates and cuvettes is 50–200 μL, and these standardized sample containers are used not only for optical OP sensors but also for most biological analyses. So, we designed a device that has circular electrodes for a larger immobilization area than our previously-reported device for larger sample volume. For comparison with other whole-cell OP sensors, the concentration of paraoxon was measured using our film, and the specific activity was calculated. The results showed that our method has 2.5 times the specific activity shown by other whole-cell OP sensors. This suggests that *E. coli*-attached SWNT film exhibits excellent performance compared to other methods. In addition, our method requires less time for cell immobilization and has better long-term stability.

We demonstrated that our microbe-attached SWNT film can be used not only for microfluidic systems, but also for 96-well microplates and cuvettes, by applying OP detection. We believe our method is optimal for enhancing performance and reducing the time required for cell immobilization in bio-application fields using microbes.

## References

[1] Cremisini C., di Sario S., Mela J., Pilloton R., Palleschi G. (1995). Evaluation of the use of free and immobilised acetylcholinesterase for paraoxon detection with an amperometric choline oxidase based biosensor. Anal. Chim. Acta.

[2] Zhang S., Zhao H., John R. (2001). Development of a quantitative relationship between inhibition percentage and both incubation time and inhibitor concentration for inhibition biosensors—Theoretical and practical considerations. Biosens. Bioelectron..

[3] Sherma J. (1993). Pesticides. Anal. Chem..

[4] Miller J.K., Lenz D.E. (2001). Development of an immunoassay for diagnosis of exposure to toxic organophosphorus compounds. J. Appl. Toxicol..

[5] Musameh M.M., Gao Y., Hickey M., Kyratzis I.L. (2012). Application of Carbon Nanotubes in the Extraction and Electrochemical Detection of Organophosphate Pesticides: A Review. Anal. Lett..

[6] Ghanem E., Raushel F.M. (2005). Detoxification of organophosphate nerve agents by bacterial phosphotriesterase. Toxicol. Appl. Pharmacol..

[7] Cho C.M.-H., Mulchandani A., Chen W. (2006). Functional analysis of organophosphorus hydrolase variants with high degradation activity towards organophosphate pesticides. Protein Eng. Des. Sel..

[8] Mello S.V., Coutures C., Leblanc R.M., Cheng T.-C., Rastogi V.K., DeFrank J.J. (2001). Interaction between organophosphorous hydrolase and paraoxon studied by surface chemistry in situ at air–water interface. Talanta.

[9] Aubert S.D., Li Y., Raushel F.M. (2004). Mechanism for the Hydrolysis of Organophosphates by the Bacterial Phosphotriesterase. Biochemistry (Mosc.).

[10] Dumas D.P., Caldwell S.R., Wild J.R., Raushel F.M. (1989). Purification and properties of the phosphotriesterase from Pseudomonas diminuta. J. Biol. Chem..

[11] Wong K.-Y., Gao J. (2007). The Reaction Mechanism of Paraoxon Hydrolysis by Phosphotriesterase from Combined QM/MM Simulations†. Biochemistry (Mosc.).

[12] Zheng F., Zhan C.-G., Ornstein R.L. (2001). Theoretical studies of reaction pathways and energy barriers for alkaline hydrolysis of phosphotriesterase substrates paraoxon and related toxic phosphofluoridate nerve agents. J. Chem. Soc. Perkin Trans 2.

[13] Singh A.K., Flounders A.W., Volponi J.V., Ashley C.S., Wally K., Schoeniger J.S. (1999). Development of sensors for direct detection of organophosphates. Part I: Immobilization, characterization and stabilization of acetylcholinesterase and organophosphate hydrolase on silica supports. Biosens. Bioelectron..

[14] Van Dyk J.S., Pletschke B. (2011). Review on the use of enzymes for the detection of organochlorine, organophosphate and carbamate pesticides in the environment. Chemosphere.

[15] Kang D.G., Lim G.-B., Cha H.J. (2005). Functional periplasmic secretion of organophosphorous hydrolase using the twin-arginine translocation pathway in Escherichia coli. J. Biotechnol..

[16] Choi J.H., Lee S.Y. (2004). Secretory and extracellular production of recombinant proteins using Escherichia coli. Appl. Microbiol. Biotechnol..

[17] Mulchandani A., Mulchandani P., Kaneva I., Chen W. (1998). Biosensor for Direct Determination of Organophosphate Nerve Agents Using Recombinant Escherichia coli with Surface-Expressed Organophosphorus Hydrolase. 1. Potentiometric Microbial Electrode. Anal. Chem..

[18] Mulchandani A., Kaneva I., Chen W. (1998). Biosensor for Direct Determination of Organophosphate Nerve Agents Using Recombinant Escherichia coli with Surface-Expressed Organophosphorus Hydrolase. 2. Fiber-Optic Microbial Biosensor. Anal. Chem..

[19] Richins R.D., Kaneva I., Mulchandani A., Chen W. (1997). Biodegradation of organophosphorus pesticides by surface-expressed organophosphorus hydrolase. Nat. Biotech..

[20] Shimazu M., Mulchandani A., Chen W. (2001). Simultaneous degradation of organophosphorus pesticides and p-nitrophenol by a genetically engineered Moraxella sp. with surface-expressed organophosphorus hydrolase. Biotechnol. Bioeng..

[21] Kim C.S., Choi B.-H., Seo J.H., Lim G., Cha H.J. (2013). Mussel adhesive protein-based whole cell array biosensor for detection of organophosphorus compounds. Biosens. Bioelectron..

[22] Kang D.G., Choi S.S., Cha H.J. (2006). Enhanced Biodegradation of Toxic Organophosphate Compounds Using Recombinant Escherichia coli with Sec Pathway-Driven Periplasmic Secretion of Organophosphorus Hydrolase. Biotechnol. Prog..

[23] Kang D.G., Kim C.S., Seo J.H., Kim I.G., Choi S.S., Ha J.H., Nam S.W., Lim G., Cha H.J. (2012). Coexpression of molecular chaperone enhances activity and export of organophosphorus hydrolase in Escherichia coli. Biotechnol. Prog..

[24] Kim C.S., Seo J.H., Kang D.G., Cha H.J. (2014). Engineered whole-cell biocatalyst-based detoxification and detection of neurotoxic organophosphate compounds. Biotechnol. Adv..

[25] Kumar J., D’Souza S.F. (2010). An optical microbial biosensor for detection of methyl parathion using Sphingomonas sp. immobilized on microplate as a reusable biocomponent. Biosens. Bioelectron..

[26] Kumar J., D’Souza S.F. (2011). Immobilization of microbial cells on inner epidermis of onion bulb scale for biosensor application. Biosens. Bioelectron..

[27] Kumar J., Jha S.K., D’Souza S.F. (2006). Optical microbial biosensor for detection of methyl parathion pesticide using Flavobacterium sp. whole cells adsorbed on glass fiber filters as disposable biocomponent. Biosens. Bioelectron..

[28] Michelini E., Roda A. (2012). Staying alive: New perspectives on cell immobilization for biosensing purposes. Anal. Bioanal. Chem..

[29] Richard J., Lewis S. (2012). Sax’s Dangerous Properties of Industrial Materials.

[30] Cassidy M.B., Lee H., Trevors J.T. (1996). Environmental applications of immobilized microbial cells: A review. J. Ind. Microbiol..

[31] Park J.K., Chang H.N. (2000). Microencapsulation of microbial cells. Biotechnol. Adv..

[32] Costerton J.W., Lewandowski Z., Caldwell D.E., Korber D.R., Lappin-Scott H.M. (1995). Microbial Biofilms. Annu. Rev. Microbiol..

[33] Kim I., An T., Choi W., Kim C.S., Cha H.J., Lim G. (2013). Site-specific immobilization of microbes using carbon nanotubes and dielectrophoretic force for microfluidic applications. RSC Adv..

[34] Kanai Y., Khalap V.R., Collins P.G., Grossman J.C. (2010). Atomistic Oxidation Mechanism of a Carbon Nanotube in Nitric Acid. Phys. Rev. Lett..

[35] Lee G.-W., Kumar S. (2005). Dispersion of Nitric Acid-Treated SWNTs in Organic Solvents and Solvent Mixtures. J. Phys. Chem. B.

[36] Marshall M.W., Popa-Nita S., Shapter J.G. (2006). Measurement of functionalised carbon nanotube carboxylic acid groups using a simple chemical process. Carbon.

[37] An T., Kim K.S., Hahn S.K., Lim G. (2010). Real-time, step-wise, electrical detection of protein molecules using dielectrophoretically aligned SWNT-film FET aptasensors. Lab. Chip.

[38] Cang-Rong J.T., Pastorin G. (2009). The influence of carbon nanotubes on enzyme activity and structure: Investigation of different immobilization procedures through enzyme kinetics and circular dichroism studies. Nanotechnology.

